# A bi-dimensional genome scan for prolificacy traits in pigs shows the existence of multiple epistatic QTL

**DOI:** 10.1186/1471-2164-10-636

**Published:** 2009-12-29

**Authors:** José L Noguera, Carmen Rodríguez, Luis Varona, Anna Tomàs, Gloria Muñoz, Oscar Ramírez, Carmen Barragán, Meritxell Arqué, Jean P Bidanel, Marcel Amills, Cristina Ovilo, Armand Sánchez

**Affiliations:** 1 Genètica i Millora Animal, IRTA-Lleida, 25198 Lleida, Spain; 2 Departamento de Mejora Genética Animal, SGIT-INIA, 28040 Madrid, Spain; 3 Departament de Ciència Animal i dels Aliments, UAB, 08193 Bellaterra, Spain; 4 INRA, UR337 Station de Génétique Quantitative et appliquée F-78350 Jouy-en-Josas, France

## Abstract

**Background:**

Prolificacy is the most important trait influencing the reproductive efficiency of pig production systems. The low heritability and sex-limited expression of prolificacy have hindered to some extent the improvement of this trait through artificial selection. Moreover, the relative contributions of additive, dominant and epistatic QTL to the genetic variance of pig prolificacy remain to be defined. In this work, we have undertaken this issue by performing one-dimensional and bi-dimensional genome scans for number of piglets born alive (NBA) and total number of piglets born (TNB) in a three generation *Iberian *by *Meishan *F_2 _intercross.

**Results:**

The one-dimensional genome scan for NBA and TNB revealed the existence of two genome-wide highly significant QTL located on *SSC13 *(*P *< 0.001) and *SSC17 *(*P *< 0.01) with effects on both traits. This relative paucity of significant results contrasted very strongly with the wide array of highly significant epistatic QTL that emerged in the bi-dimensional genome-wide scan analysis. As much as 18 epistatic QTL were found for NBA (four at *P *< 0.01 and five at *P *< 0.05) and TNB (three at *P *< 0.01 and six at *P *< 0.05), respectively. These epistatic QTL were distributed in multiple genomic regions, which covered 13 of the 18 pig autosomes, and they had small individual effects that ranged between 3 to 4% of the phenotypic variance. Different patterns of interactions (a × a, a × d, d × a and d × d) were found amongst the epistatic QTL pairs identified in the current work.

**Conclusions:**

The complex inheritance of prolificacy traits in pigs has been evidenced by identifying multiple additive (*SSC13 *and *SSC17*), dominant and epistatic QTL in an *Iberian *× *Meishan *F_2 _intercross. Our results demonstrate that a significant fraction of the phenotypic variance of swine prolificacy traits can be attributed to first-order gene-by-gene interactions emphasizing that the phenotypic effects of alleles might be strongly modulated by the genetic background where they segregate.

## Background

In the last few years, a major breakthrough in the understanding of the genetic factors that shape complex traits has been the demonstration that, in several species, a non-negligible fraction of the genetic variance is explained by epistatic interactions. The recent identification of multiple epistatic QTL controlling complex traits in mice [1-4], chickens [5,6], and in model organisms such as yeast [7] and *Drosophila melanogaster *[8,9] has been a major achievement in the understanding of the genetic nature of complex traits. In addition, the discovery that gene expression is modulated, amongst others, by a plethora of regulatory RNAs with diverse functions and properties has added a new and thick layer of complexity in the subsequent identification of the polymorphisms involved in these interactions, since many of them might reside in non-coding regions [10].

In domestic species, traits relying on reproductive physiology, such as prolificacy and fecundity, have a notable impact on the financial outcome of farming enterprises. In pigs, prolificacy is a complex trait that displays a low heritability and strong heterosis [11]. One-dimensional studies have reported the existence of several QTL affecting litter size in pigs [12-16]. However, only one of the reported QTL was significant on a genome-wide level (p < 0.05) [16], and there was a general lack of positional concordance amongst different genome scans [17]. More importantly, these QTL studies exclusively dissected the additive and dominance components of litter size, thus neglecting the analysis of epistatic interactions that, paradoxically, are expected to explain a substantial portion of genetic variation of reproductive traits [18]. In consequence, many unsolved questions concerning to the genetic architecture of pig prolificacy still remain to be answered. Which are the specific contributions of dominance and epistasis in modelling the phenotypic expression of this complex trait? If epistasis is important, which are the dimensions, geometry and intricacy of the network of interacting loci and which types of epistatic interactions are more relevant? In a cross between two inbred mice strains Peripato *et al*. [3] demonstrated the existence of eight interacting QTL that explain almost 49% of the phenotypic variance of litter size in this cross. These results highlighted the importance of non additive genetic variance as a fundamental component of prolificacy. Nevertheless, laboratory mice strains are usually bred in a very stable environment, where fluctuations are kept to a minimum, and they have been the subject of an intense process of genetic selection without parallel in any other mammal species. Moreover, mice belong to a different superorder (Euarchontoglires) than most of mammalian domestic species (Laurasiatheria), so it is reasonable to expect that in these two distantly related taxonomic groups the biology of reproduction can differ in many instances.

The relevance of the aforementioned questions led us to analyse the genetic architecture of prolificacy traits in pigs. In this way, we have performed an F_2 _intercross between two distinct European and Asian breeds, the *Iberian *and *Meishan *porcine breeds. Chinese *Meishan *is one of the most prolific pig breeds of the world being an excellent candidate population to perform these kinds of studies [19]. *Iberian *is an autochthonous Spanish breed with a very low prolificacy [20]. There is a very marked phenotypic difference for prolificacy traits between these two breeds (around 7 piglets per parity), being 14.3 the mean for the number of piglets born alive per parity of the *Meishan *breed [19] and 7.0 the mean for this trait of the *Iberian Guadyerbas *strain [20]. Interestingly, the ancestors of these breeds are assumed to have diverged at least 150,000 years before present without subsequent introgressions [21]. In consequence, it is reasonable to expect that these breeds have evolved, since then, by following independent processes of artificial selection and genetic drift, thereby establishing different adaptive epistatic genetic complexes [22]. In the current work, we have performed both a one-dimensional and a bi-dimensional genome-wide scans for prolificacy traits by employing this *Iberian *by *Meishan *F_2 _intercross as a genetic resource. Our main objective was to elucidate if epistasis makes a major contribution in shaping the phenotypic variability of prolificacy in pigs.

## Results

### Phenotypic data recorded in the F_2 _sows and linkage map

A description of the data and statistics of phenotypic records of number of piglets born alive (NBA) and total number of piglets born (TNB) in the F_2 _population is given in Table 1. The phenotypic variance was 10.24 and 9.61 for TNB and NBA, respectively. The linkage map of the 115 markers used in the QTL analyses is shown in Table 2. Marker order and distances as well as average chromosome lengths were in general agreement with other mapping projects and the USDA genome database http://www.animalgenome.org/pig/. Markers provided coverage of the 18 autosomes, with intervals between adjacent markers that were below 20 cM whenever possible. The average marker interval was 17.6 cM (sex-averaged map distance).

**Table 1:** Data structure for number of piglets born alive (NBA) and total number of piglets born (TNB) in the *Iberian *× *Meishan *F_2 _experimental population.

	Order of parity				All
		
	1	2	3	4	
N of litters	252	225	210	194	881
NBA	7.9 (3.4)^*a*^	8.3 (3.0)	8.9 (2.9)	9.2 (3.0)	8.5 (3.2)
TNB	8.7 (3.0)	8.5 (3.1)	9.3 (2.9)	9.7 (3.1)	9.1 (3.1)

^*a*^Mean (sd)

**Table 2:** Description of the markers employed for linkage analyses

*SSC*	Marker	**Pos**^1^	***SSC**^2^*	Marker	Pos	***SSC***	Marker	Pos	***SSC***	Marker	Pos
1	SW1515	0	5	SJ024	0	9	SW983	0	13	S0076	0
	ESR1	10		SWR453	44		SW21	10		SWR1008	26
	CGA	54		SW2425	55		SW911	33		SW398	48
	S0113	82		S0005	71		SW2571	69		SW2440	69
	S0151	92		SW1987	80		SW2093	95		SW769	84
	SW1828	121		IGF1	99		SW2116	130	14	SW857	0
	DBH	152		SW378	117		SW1349	149		SW1125	19
2	IGF2	0	6	MC1R	0	10	S0038	0		SW210	37
	S0141	35		SW973	22		SW1894	25		S0007	50
	SW240	49		SW1057	47		SW2195	40		SW1081	61
	SW395	65		S0087	64		S0070	52		SW1557	81
	S0226	75		SW316	87		SW1991	66		SW2515	96
	S0378	94		S0228	104		SW1626	94	15	S0355	0
	S0036	140		SW1881	119		SWR67	104		SW919	10
3	SW72	0		SW1328	153	11	S0385	0		SW1111	25
	S0206	16		SW2419	160		S0182	26		S0149	50
	S0164	33	7	S0025	0		SW2008	38		SW936	70
	S0216	64		TNFB	64		S0071	56		SW1119	100
	S0002	88		S0066	87		SW703	85	16	SW742	0
	SW349	98		SW632	117		SW2413	100		PRLR	20
4	SW2403	0		S0212	149	12	SW2490	0		SW403	30
	S0301	14		S0101	159		SW2494	10		SW2517	60
	S0001	22	8	SW2410	0		SW1307	43		S0061	88
	SW839	45		SWR1101	42		SW874	59	17	SW24	0
	S0214	63		S0017	73		SW1956	71		SW2142	14
	SW445	78		S0225	91		S0106	84		SW1920	30
	VCAM1	99		SW61	113		SWR1021	100		S0359	43
	S0097	123		BMPR1β	122					SW2431	71
									18	SW1023	0
										SW787	20
										S0120	32
										SWR414	54

^1^Pos: Position of the marker; ^2 ^*SSC*, *Sus Scrofa Chromosome*

### One dimensional genome scan for TNB and NBA

Results of the whole-genome scan using a single-QTL model for TNB and NBA are summarized in Table 3. Two genome-wide highly significant QTL were identified on *SSC13 *(*P *< 0.001) and *SSC17 *(*P *< 0.01) at similar positions for both traits. In *SSC13*, the single QTL for NBA and TNB were found at positions 50 and 55 cM, respectively (Table 3), sharing an overlapping region located between markers *SW398 *and *SW2440*. The most likely position for the QTL found on *SSC17 *was at 22 cM for both traits (Table 3).

**Table 3:** Significant single quantitative trait loci (QTL) for number of piglets born alive (NBA) and total number of piglets born (TNB)

Trait	*SSC*	Position cM (CI)	LR	Genome-wide significance level (*P*-value)	a (SE)*	d (SE)
NBA	13	50 (40-59)	24.61	< 0.001	0.71 (0.18) (Meishan)	0.69 (0.25)
	17	22 (11-42)	22.48	< 0.01	0.73 (0.19) (Iberian)	-0.82 (0.29)
TNB	13	55 (43-64)	21.93	< 0.01	0.61 (0.18) (Meishan)	0.89 (0.28)
	17	22 (12-62)	21.25	< 0.01	0.68 (0.18) (Iberian)	-0.75 (0.28)

*SSC*: *Sus Scrofa Chromosome*. cM: centimorgan. CI: confidence interval. LR: Likelihood ratio. a: additive effect. d: dominance effect. SE: standard error. * Increase in the number of piglets per copy of either *Meishan *or *Iberian *allele.

Highly significant additive and dominance effects were detected for the *SSC13 *and *SSC17 *QTL, although the direction of these effects (*Iberian vs*. *Meishan*) depended on the chromosome under consideration. For instance, the QTL on *SSC13 *for NBA increased additively by 0.71 (± 0.18) piglets per copy of the *Meishan *allele and it had a dominance effect of 0.69 (± 0.25) piglets. Conversely, the *Iberian *allele was the one associated with an increase in 0.73 (± 0.19) piglets per copy for the QTL on *SSC17*. Moreover, this QTL on *SSC17 *displayed a negative dominance effect (-0.82 ± 0.29). We estimated the degree of dominance as the ratio d/a between the estimated dominance (d) and the absolute value of the additive effect (a). Values of d/a larger than unity corresponds to overdominance, while a d/a ratio between 0 and 1 represents partial dominance. In both cases (QTL on *SSC13 *and *SSC17*), the estimated d/a values were consistent with a complete dominance situation. Similar values of additive and dominance effects were obtained for the TNB QTL on *SSC13 *and *SSC17 *(Table 3), a result that is not surprising since these two traits are highly correlated. Besides, the proportion of phenotypic variance explained by these two single QTL detected on *SSC13 *and *SSC17 *ranged from 2% to 3% for both TNB and NBA.

### Bi-dimensional genome scans for NBA and TNB

#### Identification of multiple interacting QTL for NBA and TNB

Results from the bi-dimensional genome scan for NBA are shown in Table 4. Four bi-dimensional genome-wide highly significant (*P *< 0.01) and five significant (*P *< 0.05) epistatic interactions between QTL were found. We confirmed that all the observed epistatic interactions were consistently detected across families rather than being the consequence of a single sire effect, a feature that is particularly important when the number of founder males is moderate or even small. The results obtained through the likelihood ratio test were further confirmed by using other approaches. First, a false discovery rate (FDR) was calculated based on the nominal *P*-values every 30 cM, with a result of 0.018 for a *P*-value lower than 0.001. Moreover, parametric bootstrapping confirmed the significance of the results.

**Table 4:** Results of a bi-dimensional genome scan for number of piglets born alive (NBA)

*SSC_1_*	*Position_1 _cM (CI)*	*SSC_2_*	*Position_2 _cM (CI)*	***LR***	***(P-value)***	*a_1 _(SE)*	*d_1 _(SE)*	***a_2 _(SE)***	***d_2 _(SE)***	***a × a (SE)***	***a × d (SE)***	***d × a (SE)***	***d × d (SE)***
1	153 (146-153)	6	55 (50-60)	27.34	<0.05								3.34 (0.72)
1	76 (69-84)	7	107 (101-116)	31.43	<0.01	-0.53 (0.18)		0.73 (0.31)	0.85 (0.38)			2.61 (0.57)	3.83 (0.97)
5	66 (59-73)	18	11 (2-20)	27.97	<0.05			0.71 (0.24)		-1.49 (0.40)		1.75 (0.47)	
6	59 (54-64)	7	28 (12-37)	31.25	<0.01							2.88 (0.69)	6.20 (1.51)
6	4 (1-10)	14	29 (25-36)	30.27	<0.01			0.61 (0.25)				1.63 (0.42)	2.89 (0.74)
8	92 (88-94)	10	87 (81-103)	28.59	<0.05				-0.82 (0.32)			-1.50 (0.44)	-2.86 (0.75)
9	4 (1-7)	13	73 (66-82)	30.58	<0.01			-0.75 (0.22)		0.87 (0.35)			-3.31 (0.65)
10	99 (89-104)	15	3 (1-8)	29.41	<0.05					1.25 (0.34)	-1.25 (0.36)		-1.30 (0.62)
12	11 (9-18)	12	89 (74-96)	28.82	<0.05	0.34 (0.17)		0.90 (0.22)		-1.36 (0.30)	0.83 (0.39)		2.45 (0.60)

*SSC*_1 _and Position_1_: Chromosome and position, in centimorgans (cM), of the first location. *SSC*_2 _and Position _2_: Chromosome and position, in centimorgans (cM), of the second location. CI: Confidence interval. LR, Likelihood ratio of contrast of the model with and without epistasis. a_1 _additive effect of the first location. a_2 _additive effect of the second location d_1 _dominance effect of the first location. d_2 _dominance effect of the second location. Epistasis type: a × a: additive by additive effect; a × d: additive by dominance effect; d × a: dominance by additive effect: d × d, dominance by dominance effect. SE: standard error. Only significant values of a, d, and their interactions are shown.

**Figure 1:**
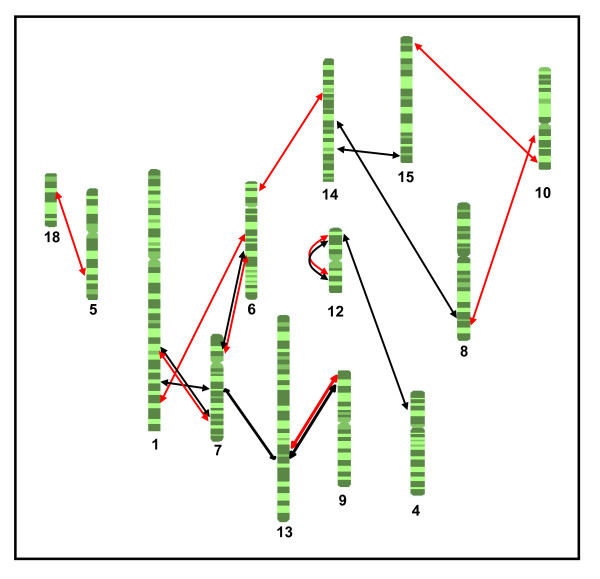
**Network representation of the epistatic QTL interactions in thirteen pig chromosomes (*SSC*) for prolificacy traits NBA (red arrows) and TNB (black arrows)**.

A graphical overview of the epistatic interactions for NBA (red arrows) is shown in Figure 1. As much as twelve of the 18 pig autosomes (1, 5, 6, 7, 8, 9, 10, 12, 13, 14, 15 and 18) were involved in these interactions, forming a complex network with a non-radial geometry. This means that a specific region did not interact simultaneously with multiple loci, but with a very limited number of them (usually interactions were one to one). For example, the *SSC12 *region located at 11 cM, interacted significantly with another *SSC12 *region at 89 cM (Figure 1; Table 4). Similarly, two non-overlapping *SSC6 *QTL regions showed epistatic interactions, one of them with QTL on *SSC1 *and *SSC7 *(*SSC6*, 54-69 cM) and another one with *SSC14 *(*SSC6*, 1-10 cM). As shown in Figure 1, other pig chromosomes exhibiting more than one significant interacting QTL were *SSC1 *(at positions 76 and 153 cM respectively), and *SSC7 *(at positions 28 cM, and 107 cM). An interesting feature of our analysis was that the highly significant NBA QTL identified in the one-dimensional genome scan (*SSC13 *at 50 cM and *SSC17 *at 22 cM) did not show any significant epistatic interaction with other regions across the genome, meaning that its mode of action is purely additive. In contrast, a NBA QTL found on another region of *SSC13 *(73 cM) had significant epistatic interactions with a QTL located at position 4 cM of *SSC9*. Similarly, in mice, Peripato et al. [3] identified two significant QTL for litter size in a one-dimensional genome scan (chromosomes 7 and 12) that did not emerge in the bi-dimensional analysis (chromosomes 2, 4, 5, 11, 14, 15 and 18). In the light of these results and ours, we could conclude that there is a low concordance between the QTL identified in one- and bi-dimensional genome scans. This means that, in general, the additive and epistatic components of prolificacy traits encompass different sets of genes.

With regard to the bi-dimensional genome-wide scan for TNB, we found three genome-wide highly significant (*P *< 0.01) and six significant (*P *< 0.05) epistatic interactions (Table 5; Figure 1). Thirteen out of the 18 pig autosomes were involved in the epistatic QTL interactions for both traits. However, the network of interacting QTL for TNB was not identical to the one reported for NBA. In this sense, only around one third of the QTL epistatic interactions detected in the current study were coincident in both traits. This was an unexpected result since NBA and TNB display a high genetic correlation (r_g_~ 0.9) and they are expected to share a similar genetic architecture [11]. Moreover, the number of chromosomes displaying multiple interactions was higher for TNB (*SSC1*, *7*, *12*, *13 *and *14*) than for NBA. Notably, four epistatic QTL on *SSC7 *showed significant interactions with QTL located on *SSC6 *(60 cM), *SSC13 *(77 cM) and *SSC1 *(79 cM and 139 cM, respectively). Other epistatic interactions involved two overlapping regions of *SSC13 *with one region of *SSC7 *and one *SSC9 QTL*, a feature that demonstrates the remarkable complexity and intricacy of these networks. Finally, it is worth mentioning that if we would have assumed a type 1 error α = 0.10, which corresponds to a genome-wide critical value of 25.14 for the LR test, we would have been able to detect 11 and 9 additional epistatic interactions between QTL across the genome for NBA and TNB, respectively (results not shown).

**Table 5:** Results of a bi-dimensional genome scan for total number of piglets born (TNB)

*SSC_1_*	*Position_1 _cM (CI)*	*SSC_2_*	*Position_2 _cM (CI)*	***LR***	***(P-value)***	***a_1 _(SE)***	***d_1 _(SE)***	***a_2 _(SE)***	***d_2 _(SE)***	***a × a (SE)***	***a × d (SE)***	***d × a (SE)***	***d × d (SE)***
1	79 (72-84)	7	107 (100-116)	31.70	<0.01	-0.55 (0.17)	-0.68 (0.29)	0.68 (0.29)				2.27 (0.49)	3.29 (0.85)
1	139 (132-147)	7	89 (84-96)	28.47	<0.05				0.63 (0.31)		-2.10 (0.54)	2.08 (0.60)	2.18 (1.04)
4	23 (21-26)	12	5 (1-10)	27.45	<0.05		0.65 (0.25)			0.80 (0.33)		-0.92 (0.35)	-2.61 (0.57)
6	60 (55-69)	7	24 (12-33)	35.01	<0.01							2.47 (0.62)	5.29 (1.30)
7	70 (46-77)	13	77 (73-81)	28.41	<0.05			-1.03 (0.26)	0.84 (0.28)	1.08 (0.39)	-1.56 (0.50)	-1.09 (0.46)	-3.14 (0.82)
8	83 (76-89)	14	58 (54-63)	29.63	<0.05						2.29 (0.46)		
9	4 (1-10)	13	74 (63-81)	26.99	<0.05			-0.70 (0.21)	0.63 (0.27)	0.72 (0.34)			-2.98 (0.64)
12	11 (8-19)	12	86 (77-93)	32.10	<0.01			1.01 (0.20)		-1.38 (0.27)	0.73 (0.34)		2.00 (0.52)
14	89 (80-97)	15	100 (96-101)	28.30	<0.05					0.79 (0.34)	-1.14 (0.40)	-1.20 (0.41)	-2.62 (0.69)

*SSC*_1 _and Position_*1*_: Chromosome and position, in centimorgans (cM), of the first QTL location. *SSC*_2 _and Position_*2*_: Chromosome and position, of the second QTL location. CI: Confidence interval interval. LR, Likelihood ratio of contrast of the models with and without epistasis. a_1_: additive effect of the first location. a_2_: additive effect of the second location. d_1_: dominance effect of the first location. d_2_: dominance effect of the second location. a × a: additive by additive effect. a × d: additive by dominance effect. d × a: dominance by additive effect. d × d: dominance by dominance effect. SE: standard error. Only significant values of a, d, and their interactions are shown.

#### Partition of the phenotypic variance explained by epistatic QTL

We estimated the proportion of the phenotypic variance explained by epistatic genetic components for each TNB and NBA significant epistatic QTL pair. This contribution to the total phenotypic variance ranged from 3.26% (*SSC14-SSC15*) to 4.04% (*SSC12-SSC12*) for TNB and from 3.10% (*SSC1-SSC6*) to 3.62% (*SSC9-SSC13*) for NBA. The relative contribution of epistasis to the total phenotypic variance was estimated by adding the estimates of the partial epistatic effects of each epistatic interaction. The total phenotypic variance explained by the joint genetic effects of all epistatic QTL pairs for NBA and TNB was 37.6% and 42.4%, respectively. Nevertheless, we would like to mention that this approach might lead to an overestimation of the epistatic contribution because the models employed in our analysis are exclusive [23]. We have calculated the repeatability with a standard animal model, resulting in an estimated value of 0.27 for both TNB and NBA. This value should be considered as the limit of the total variance explained by genetic effects. This result evidences that our estimates of epistatic contributions are clearly overestimated but, in spite of this drawback, we think it is reasonable to assume that a significant proportion of the phenotypic variance of prolificacy is explained by the joint genetic effects of epistatic QTL.

#### Classification of epistatic effects for NBA and TNB

**Figure 2:**
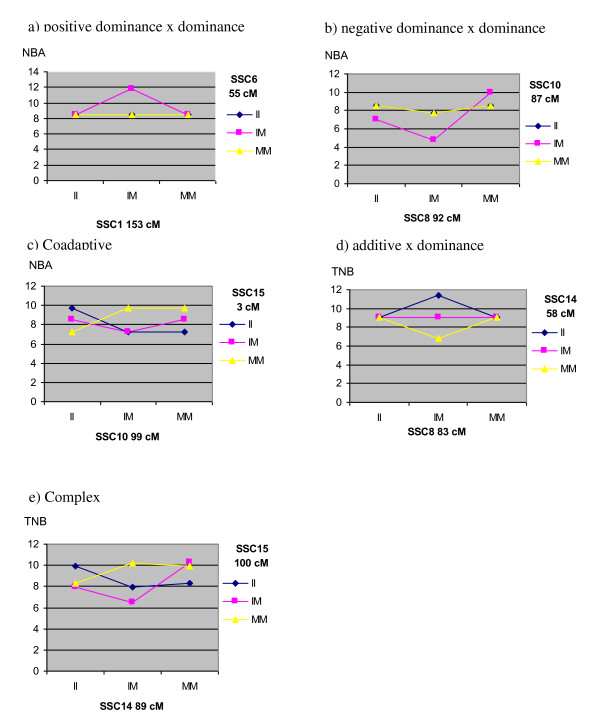
**Genotypic values expected for each epistatic pattern detected in the experimental F_2 _*Iberian *× *Meishan *intercross. *SSC*: *Sus Scrofa Chromosome***. Genotypes are shown as II (Iberian/Iberian homozygote), IM (Iberian/Meishan heterozygote), MM (Meishan/Meishan homozygote).

All four forms of epistasis (*a *× *a*, *a *× *d*, *d *× *a *and *d *× *d*) were detected among the significant epistatic pairs. In total, 9 *a *× *a*, 7 *a *× *d*, 11 *d *× *a*, and 16 *d *× *d *significant interactions were detected (Tables 4 and 5). For the *SSC1-SSC6 *(NBA) and *SSC8-SSC14 *(TNB) epistatic pairs, only one type of epistasis was significant (*d *× *d*, and *a *× *d*, respectively); whereas, the remaining pairs presented two or more types of epistasis, leading to more complex patterns of interactions. We plotted the genotypic values expected for each genotypic class (Figure 2), thus identifying 'dominance-by-dominance' and 'coadaptive' patterns of epistasis as described by Carlborg and Haley [18] and Carlborg *et al*. [4]. The 'dominance-by-dominance' pattern of epistasis was found in several epistatic QTL pairs and corresponded to interactions were a significant *d *× *d *effect was found. In positive *d *× *d *interactions (Figure 2a), the genotypic value of double heterozygotes I_1_M_1_-I_2_M_2_, (being I = *Iberian *alleles and M = *Meishan *alleles for loci 1 and 2) was superior to that of simple heterozygotes (I_1_M_1_-I_2_I_2_, I_1_M_1_-M_2_M_2_, I_1_I_1_-I_2_M_2_, and M_1_M_1_-I_2_M_2_); a pattern that was reversed in negative *d *× *d *interactions (Figure 2b).

According to Carlborg and Haley [18], the 'coadaptive' type of interaction occurs when positive *a *× *a *interaction effect is significant. This form of epistasis leads to enhanced performance of parental double homozygotes (I_1_I_1_-I_2_I_2 _and M_1_M_1_-M_2_M_2_) in comparison to the hybrid double homozygotes (I_1_I_1_-M_2_M_2 _or M_1_M_1_-I_2_I_2_). As an example, we found this specific pattern of epistasis in the NBA epistatic QTL pair *SSC10-SSC15 *(Figure 2c).

Additionally, we found another epistatic pattern where only significant *a *× *d *and/or *d *× *a *effects were present. These pairs of interacting QTL were included in a category that we named 'additive-by-dominance' epistasis because *a *× *d *and *d *× *a *interactions showed exactly the same pattern. In positive 'additive-by-dominance' interactions, one locus is overdominant, neutral, or underdominant, depending on the genotype at the second locus. Conversely, the second locus is additive, with the favoured allele being dependent on the genotype at the first locus. In negative 'additive-by-dominance' interactions, the genotypic values of homozygotes at one locus (_._I_2_I_2 _and _._M_2_M_2_) deviate from what is expectable under an additive model only when the second locus is heterozygous (I_1_M_1_-I_2_I_2 _and I_1_M_1_-M_2_M_2_, Figure 2d).

Finally, we considered as a separate category those QTL pairs that could not be classified into any of the preceding epistasis models. The main common feature of QTL pairs gathered in this group was that they all had a significant *a *× *d *or *d *× *a *epistatic term together with significant *a *× *a *and/or *d *× *d *terms, leading to 'complex' patterns of interactions (Figure 2e). These complex patterns might be explained by the existence of higher order interactions involving more than two loci or simply because small sample size for certain genotypic classes might lead to inaccurate estimates of the epistatic effects.

## Discussion

### Identification of genome-wide significant QTL with additive and dominant effects on pig prolificacy

In the single QTL analyses, we found two genome-wide highly significant QTL located on *SSC13 *and *SSC17 *affecting NBA and TNB. So far, and to the best of our knowledge, only one study has described a genome-wide significant QTL (P < 0.05) detected at 88 cM on *SSC15 *for NBA [16]. In the current work, the mode of gene action strongly differed amongst QTL. For instance, the additive effect detected for the *SSC13 *QTL stemmed from the *Meishan *breed, and determined an increase of 0.71 and 0.61 piglets for NBA and TNB, respectively. This result is in agreement with the phenotypic differences observed between the purebred parental lines. Conversely, beneficial alleles for the QTL found on *SSC17 *appeared to derive from the *Iberian *breed, with increases of 0.73 and 0.68 piglets for NBA and TNB, respectively. These cryptic *Iberian *QTL alleles, which increase prolificacy, provide a compelling example of the complexity of the genetic architecture of these traits. In addition, significant dominance effects were also detected for the two mentioned QTL; however, the direction of the effects was complete dominance for the QTL on *SSC13*, and recessivity for the QTL at *SSC17*.

Our results confirm the existence of several previously reported suggestive QTL for litter size in pigs. De Koning *et al*. [13] described one suggestive QTL for TNB on *SSC17 *(43 cM) in an F_2 _cross between *Meishan *and commercial *Dutch *lines. Bidanel *et al*. [24] reported a QTL for ovulation rate on SSC13 with a similar location (46 cM) and effect (favourable dominant effect of the *Meishan *allele of 0.7 ova shed). The experiment of Bidanel *et al*. [24] involved an F_2 _cross between the same *Meishan *population employed in our study and a *Large White *population. Moreover, Cassady *et al*. [12] described a QTL for number of stillborn piglets on *SSC13 *(101 cM), although its location is relatively distant to the one reported by us (50-55 cM). Other QTL for litter traits have been described on *SSC5*, *SSC6*, *SSC7*, *SSC8*, *SSC11*, *SSC14*, *SSC16 *and *SSC18 *[12-15,25]. However, and as mentioned above, the majority of QTL reported in previous studies none reached the genome-wide significant threshold while two QTL identified in our study did. The most likely explanation for this discrepancy relies on the fact that most of previous experiments involved crosses between the *Meishan *breed and other standard European pig populations, such as *Large White *pigs, which have been shown to differ in only three to five piglets born [26]. Moreover, standard European breeds were strongly introgressed with Chinese breeds in the 18-19^th ^centuries and, in consequence, they might not completely fulfil the assumptions and requirements of the F_2 _intercross design. Conversely, the *Meishan *and *Iberian *breeds are highly divergent both at the phenotypic (about 7 piglets per parity) and genetic levels. In this way, phylogenetic studies carried out with mitochondrial DNA have revealed that the *Iberian *breed has never been introgressed with *Asian *alleles [21]. Finally, the number of available reproductive records was much larger in our experiment (881 experimental data) than in previous studies (between 200 and 400 experimental data) [12-15,25].

### Phenotypic variation of prolificacy traits is affected by a complex network of epistatic QTL

The statistical evidence suggesting that epistasis might be an important component of reproductive traits in mice [3] led us to perform a bi-dimensional genome scan for NBA and TNB. This analysis allowed us to demonstrate that phenotypic variation of these traits can be strongly influenced by a complex network of interacting loci. In this sense, this is the first study that shows genome-wide significant epistatic QTL affecting prolificacy in pigs. After implementing the highly stringent Bonferroni correction for multiple testing, as much as seven QTL interactions remained significant at the 1% bi-dimensional genome-wide level, whereas 11 were significant at the 5% genome-wide level. Rather than identifying one or a few master regions interacting with multiple loci (which would have yielded a star-like geometry of interactions), we found that most chromosomal regions interacted with a single counterpart. However, almost all chromosomes in which significant epistatic QTL were found participated in more than one interaction. Five of the significant epistatic interactions detected had pleiotropic effects on both TNB and NBA traits, whereas five were only related to TNB and three to NBA. These surprising differences for two highly genetically correlated traits [27], might be interpreted in the light of the statistical values of the Likelihood ratio obtained in the contrast between models and the stringent bi-dimensional genome-wide threshold assumed. For instance, the chromosome pair *SSC10-SSC15 *reached an LR value of 29.41 (p < 0.05) for NBA and an LR value of 22.33 ("no significant") for TNB. Alternatively, there might be a biological meaning behind the specific differences observed in the geometry of the NBA and TNB networks, thus indicating that although similar metabolic and physiological pathways may be implicated in the regulation of both traits, other mechanisms may be operating independently.

### Epistatic QTL for pig prolificacy display different types of interactions

This genetic dissection of the epistatic component of prolificacy in pigs was completed with an analysis of the types of interactions detected in the bi-dimensional genome-wide scan. All the epistatic QTL had at least one significant type of interaction (see tables 4 and 5). In total, nine pairs showed additive by additive (*a *× *a*) epistasis, which in certain circumstances can have a 'nullification effect' because epistasis might cancel out the effects of individual loci at intermediate frequencies, making difficult to detect them in a conventional one-dimensional genome scan [28]. Furthermore, seven pairs showed additive by dominance (*a *× *d*) epistasis, eleven pairs showed dominance by additive (*d *× *a*) epistasis, and sixteen pairs showed dominance by dominance (*d *× *d*) epistasis. All these forms of epistasis contribute to heterosis [29]. It has been widely supported that heterosis plays an important role in the genetic architecture of reproductive traits [11,12].

Several patterns of interactions among genotypes were identified and classified according to Carlborg and Haley [18] and Carlborg *et al*. [4]. Eight pairs were classified as 'dominance-by-dominance' and two pairs as 'coadaptive' epistasis. The remaining QTL showed different patterns of epistasis from those previously described in the literature and were, therefore, grouped into two additional categories that we named 'additive-by-dominance' and 'complex' epistasis. 'dominance-by-dominance' epistasis leads to a deviated performance for the double heterozygotes compared to single heterozygotes. Among the *d *× *d *interactions found in our study, six had positive sign (Figure 2a) and two had negative sign (Figure 2b). In positive *d *× *d *interactions, the genotypic value of the double homozygotes is superior to the simple heterozygotes. Conversely, this pattern is reversed when the sign is negative. Coadaptive epistasis occurs when double homozygotes from the same parental line show enhanced performance [18]. As mentioned above, we only found two QTL pairs which displayed this form of epistasis (Figure 2c). Coadaptive epistasis is fundamental to interpret post-zygotic reproductive isolation [30-32]. In each parental population, selection may have acted leading to fixation of different alleles at the relevant loci regulating prolificacy in a way that statistical epistasis is not apparent in either population. Second-generation hybrids (F_2_) exhibit combination of alleles at different loci that were not present in any of the parental breeds, leading to the disruption of "co-adapted" gene pools and the appearance of new phenotypes.

The third category included two pairs where only *a *× *d *and *d *× *a *effects were significant. We called this group 'additive-by-dominance' because the *a *× *d *and *d *× *a *interactions show the same patterns but with the roles of the loci reversed (Figure 2d). Finally, we found QTL pairs which show 'complex' patterns of interactions characterised by having a significant *a *× *d *or *d *× *a *term together with significant *a *× *a *and/or *d *× *d *(Figure 2e). These interactions yielded complex patterns in which the genotypic value for a given genotype at one locus drastically changes depending on the genotype at the second locus.

## Conclusions

In summary, the bi-dimensional genome scan of an *Iberian *× *Meishan *F_2 _intercross has allowed to demonstrate that the genetic architecture of pig reproduction is mostly built as a complex network of interacting genes rather than being explained by the sum of the additive effects of a yet to be defined number of loci. Individual epistatic loci have moderate effects on the phenotypic variance of prolificacy and they are distributed in multiple chromosomal locations. Moreover, they display several types of interactions that sometimes cannot be easily ascribed to well defined models, thus suggesting the existence of additional interacting loci. In the next years, the fine mapping and identification of the causal mutations that explain the segregation of epistatic QTL in pigs will be a daunting but fascinating task that will likely unveil many of the secrets that underlie the biological grounds of complex traits.

## Methods

### Experimental design and phenotypic data

A three-generation F_2 _intercross between *Iberian *and *Meishan *pig breeds was generated to map prolificacy QTL. Eighteen *Meishan *sows were randomly mated by artificial insemination with three *Iberian *boars (*Guadyerbas *line) to produce the F_1 _progeny in the INRA GEPA experimental unit (Surgères, France). This F_1 _offspring was purchased and transferred to NOVA GENÈTICA S. A. experimental farm (Lleida, Spain) after weaning at 22-25 days of age. At sexual maturity, eight F_1 _boars and 97 F_1 _sows were randomly selected to obtain an F_2 _progeny. The F_1 _sows produced only one litter and were slaughtered after weaning. In total, 255 F_2 _reproductive sows were randomly selected and mated to unrelated boars. They produced a total of 881 litters, i.e. 3.45 parities per F_2 _sow on average. The number of piglets born alive (NBA) and the total number of piglets born (TNB) were recorded at farrowing. Animals were managed under standard intensive conditions; in all cases, reproduction was carried out by artificial insemination. Protocols were approved by the Ethical and Animal Care Committee at IRTA.

### Microsatellite and single nucleotide polymorphism genotyping

DNA was extracted from either frozen blood or tail tissue using commercial protocols (Gentra Systems, Minneapolis). Purebred grandparents, F_1 _breeding pigs and the 255 F_2 _sows were genotyped for 115 markers: 109 microsatellites and 6 single nucleotide polymorphisms (SNP). Microsatellite loci were chosen based on their ease of scoring, the absence of null alleles, their genomic location and their informativeness. Microsatellite PCR products were analyzed with the Genescan 3.7 software (Applied Biosystems, Warrington, UK) in an ABI PRISM 3100 Genetic Analyzer (Applied Biosystems). Single nucleotide polymorphisms markers in DBH, BMPR1β, PRLR and VCAM1 genes were analyzed with the SNapSHOT ddNTP primer extension multiplex kit (Applied Biosystems, Warrington, UK) [33-36]. Moreover, two polymerase chain reaction restriction fragment length polymorphism (PCR-RFLP) markers were analysed: the MC1R gene G/A283 SNP was genotyped by PCR-RFLP with NspI [37]. The PvuII estrogen receptor 1 polymorphism was genotyped following Short et al. [38]. Linkage analysis was carried out by using the 'build' option of the CRI-MAP 2.4 program [39].

### Statistical analysis

NBA was considered to be the same trait across all parities and the same criterion was applied to TNB. Two statistical models were used to analyze the experimental data. The first model was a one-dimensional QTL mapping performed using a regression approach [40], based on the following mixed model:(1)

where *y*_*ijk *_was the *ijk*^*th *^observation for NBA or TNB, *H*_*i *_was the *i*^*th *^year-season fixed effect, *O*_*j *_was the *j*^*th *^order of parity fixed effect, *u*_*k *_was the random polygenic effect of the *k*^*th *^individual, *p*_*k *_was the random permanent environmental effects for the *k*^*th *^individual, *a *was the QTL additive effect, *d *was the dominance QTL effect and *e*_*ijkl *_was the random residual term; *c*_*a *_= *pr*(*QQ*) - *pr*(*qq*) and *c*_*d *_= 0.5*pr*(*Qq*) - 0.5(*pr*(*QQ*) + *pr*(*qq*)), where *pr(QQ) *was the probability of being homozygous of *Iberian *origin, *pr(qq) *was the probability of being homozygous of *Meishan *origin and *pr(Qq) *was the probability of being heterozygous. For computational reasons, heritability (h^2^) and percentage of permanent environmental effect (p^2^) were assumed to be known. They were obtained from the posterior mode of a previous Bayesian analysis. Estimates for h^2 ^and p^2 ^were 0.22 and 0.05, respectively. The analyses were performed at every centimorgan along the 2,017 cM of the 18 autosomes, by means of a likelihood ratio test (LR) comparing the models with and without the QTL effects. Nominal *P*-values were calculated assuming a chi-squared distribution of the LR test. Yet, nominal significance levels cannot be used directly due to the large number of tests performed. Hence, genome-wide significance levels were calculated using a Bonferroni correction and assuming independence between statistical tests every 30 cM. The genome-wide critical values of LR test for level of significance associated with type I errors α = 0.10, 0.05, 0.01 and 0.001 were 13.36, 15.13, 18.27 and 23.22, respectively.

With regard to the two-QTL analyses, two different models were employed. The first model included the effects of two non interacting QTL. The statistical mixed model was:(2)

where *a*_1 _and *a*_2 _were the additive effects, *d*_1 _and *d*_2 _were the dominance effects for QTL 1 and 2, respectively. The coefficients *c*_*a*1_, *c*_*d*1_, *c*_*a*2 _and *c*_*d*2 _were calculated as before for locations 1 and 2.

The second model allowed for epistasis, i.e.:(3)

where *I*_*a *× *a*_, *I*_*a *× *d*_, *I*_*d *× *a *_and *I*_*d *× *d *_were the additive × additive, additive × dominance, dominance × additive and dominance × dominance epistatic interaction effects, respectively; *c*_*a *× *a*_, *c*_*a *× *d*_, *c*_*d *× *a *_and *c*_*d *× *d *_were the regression coefficients calculated following Cockerham's model for epistatic interactions [41,42], i.e.:

The two-QTL analyses were performed using a full bi-dimensional genome scan. LR-tests comparing the models with and without the epistatic interaction effects (model 3 *vs *model 2) were computed at 1 cM intervals along the 2,017 cM of the 18 autosomes for each of the two QTL, leading to a total of 2,069,595 regression analyses for both NBA and TNB. The values of h^2 ^and p^2 ^used in this analysis were identical to those considered in model 1. The statistical contrast between models for evidence of epistasis was carried out using an LR test with 4 degrees of freedom in the numerator. As before, bi-dimensional genome-wide levels of significance were calculated using a Bonferroni correction assuming statistical independence every 30 cM. The genome-wide critical values of LR test for level of significance associated with type I errors α = 0.10, 0.05, 0.01 and 0.001 were 25.14, 26.68, 30.17 and 35.06 respectively. Confidence intervals for QTL location were calculated using the Likelihood drop method [43]. Finally, we calculated the expected values of the nine genotypic classes using the Cockerham's F_2_-metric model [42]. In addition, statistical significance was independently assessed by using an approach based on the False Discovery Rate -FDR- [44], that was calculated based on nominal *P*-values every 30 cM, as well as by employing a parametric bootstrap method [45].

## Authors' contributions

JLN conceived and led the project, supervised its execution, participated in the design of the study, drafted and finalized the manuscript. CR participated in the design and coordination of the study, obtained and provided Iberian semen, performs linkage analysis, and helped to revise the manuscript. LV participated in the design of the study, developed the analysis programs, performed the statistical analysis and helped to revise the manuscript. AT has carried out part of the molecular genotyping tasks and has has been involved in drafting and revising critically the manuscript. GM performed part of the molecular genotyping tasks. OR performed part of the molecular genotyping tasks. CB has performed part of the microsatellite genotyping. MA (IRTA) participated in the experimental protocol and data collection. JPB has provided Meishan sows (INRA) to obtain F1 animals, and participated in the discussion of the results and helped to revised the manuscript. MA (UAB) has been involved in drafting the manuscript and revising it critically. CO, was responsible of DNA extraction and the selection of markers to genotype and helped to perform linkage analysis. AS participated in the design and coordination of the study and helped to draft the manuscript.

All authors read and approved the final manuscript.
